# Supplementary material for: A Novel 3-Gene Signature for Identifying COVID-19 Patients Based on Bioinformatics and Machine Learning
Source: Genes (Basel). 2022 Sep 8;13(9):1602. doi: 10.3390/genes13091602 (PMC9498787; doi:10.3390/genes13091602)
Supplement: Supplementary file 1 [file genes-13-01602-s001.zip › Supplementary table and figures.pdf]

Table S1. The findings from recent state of the art.

Title	Dataset	Methods Used	Evaluation Metrics	Research Challenges
m6A Regulator-Mediated Methylation Modification Patterns and Characteristics of Immunity in Blood Leukocytes of COVID-19 Patients	GSE157103	Limma, GSVA, CIBERSORT, PCA, LASSO	true-positive fraction, false-positive fraction	To reveal m6A methylation modification of leukocyte response to viral infection in COVID-19
XGBoost-Based Feature Learning Method for Mining COVID-19 Novel Diagnostic Markers	GSE152075	edgR, XGBoost, MARS, KNN, SVM, MLP, RF, PCA, GO, KEGG	feature importance ranking, recursive elimination, IFS method	To identify some potential biomarkers for COVID-19 through XGBoost algorithm
m6A Regulator-Mediated Methylation Modification Patterns and Characteristics in COVID-19 Patients	GSE157103	Limma, RF, SVM, GO, PCA, ssGSEA	sensitivity, 1-specificity	To investigate the potential roles of m6A regulators in COVID-19
Integrated COVID-19 Predictor: Differential expression analysis to reveal potential biomarkers and prediction of coronavirus using RNA-Seq profile data	GSE152641	Limma, SVM, kNN, Naïve Bayes, Random Forest, Decision Tree	classification, accuracy, sensitivity, specificity, precision, FPR, NPV, RMC, F1, AUC	To develop a COVID-19 predictor with high predictive value

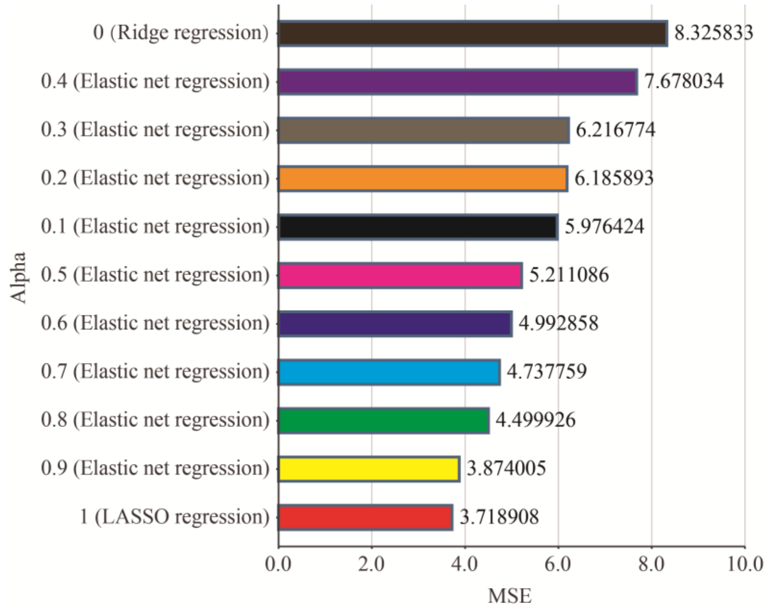

Figure S1. Comparison of LASSO regression, ridge regression, and elastic net regression for identifying COVID-19.

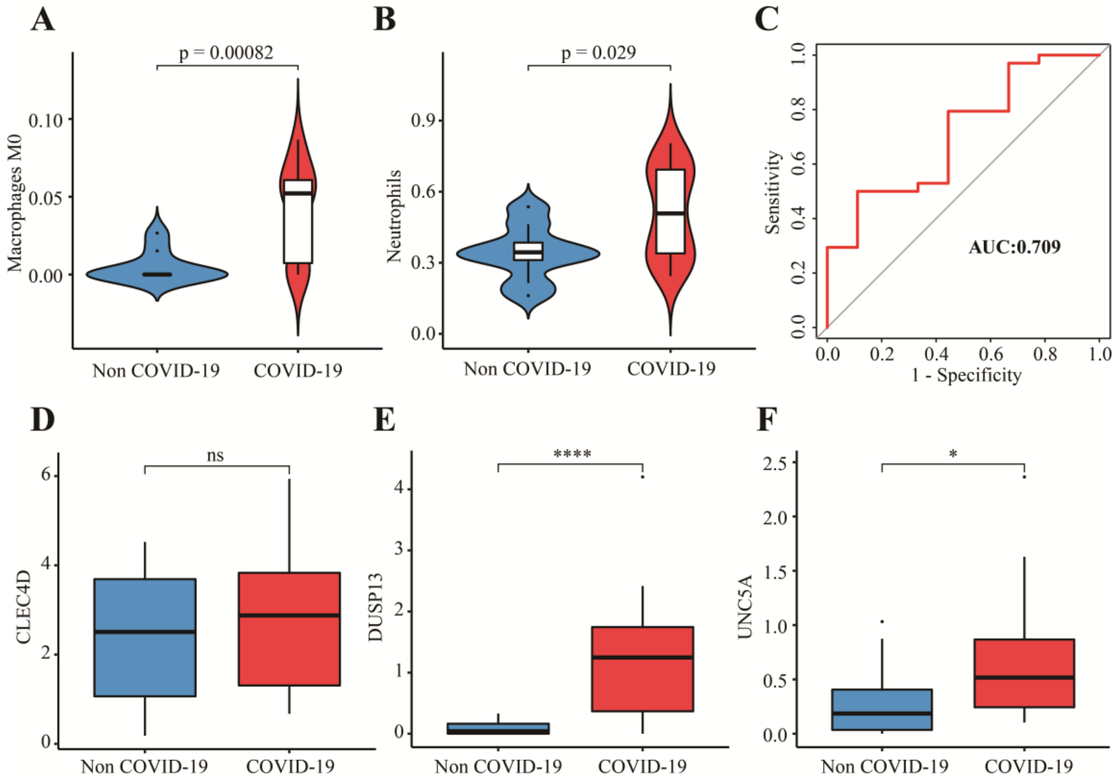

Figure S2. Validation of two important immune cells and 3-gene signature in GSE196822. (**A**) Comparison of M0 macrophages between COVID-19 and non-COVID-19 groups. (**B**) Comparison of Neutrophils between COVID-19 and non-COVID-19 groups.(**C**)The AUC for prediction of outcome. (**D-F**) The expressions of CLEC4D, DUSP13 and UNC5A between COVID-19 and non-COVID-19 groups. Data in (**D-F**) were analyzed by Wilcoxon test; ns, no significance;*$p < 0.05$,**$p < 0.01$, ***$p < 0.001$ and ****$p < 0.0001$.
