# Supplementary figures and images for: A Novel 3-Gene Signature for Identifying COVID-19 Patients Based on Bioinformatics and Machine Learning
Source: Genes (Basel). 2022 Sep 8;13(9):1602. doi: 10.3390/genes13091602 (PMC9498787; doi:10.3390/genes13091602)

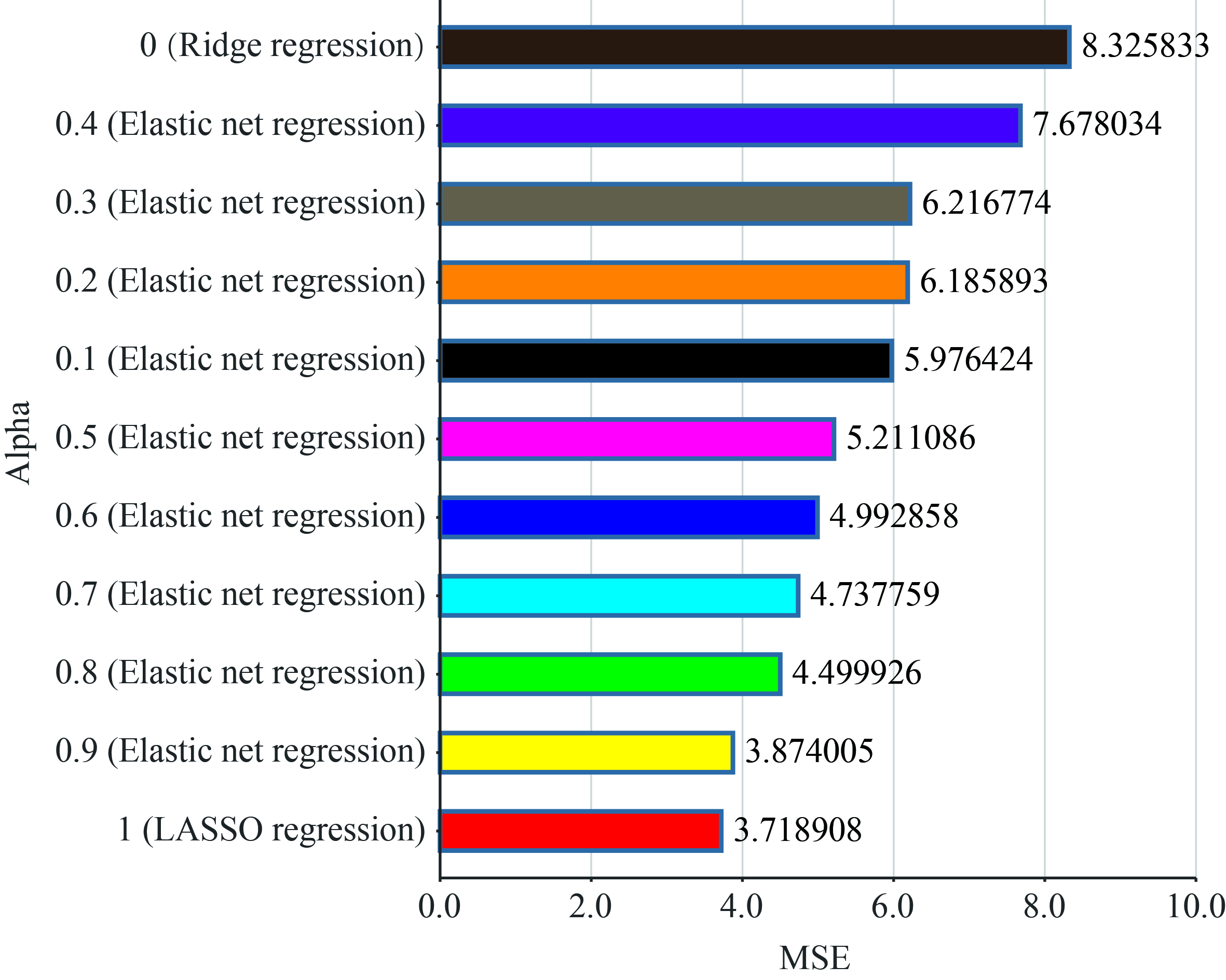

Supplement: Supplementary file 1 [file genes-13-01602-s001.zip › Figure S1.tif]

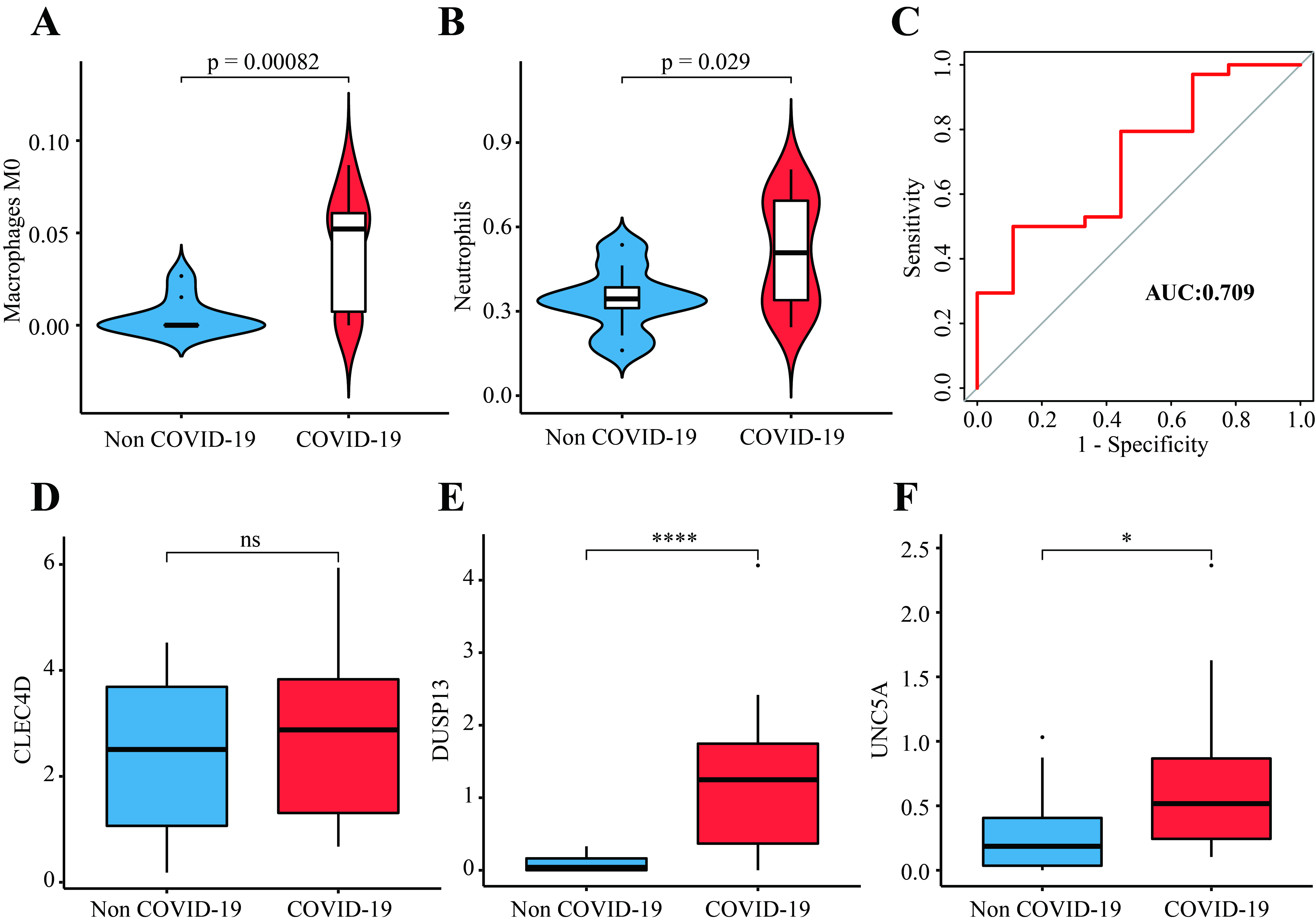

Supplement: Supplementary file 1 [file genes-13-01602-s001.zip › Figure S2.tif]
